# Orthogonal high-density mapping with ventricular tachycardia isthmus analysis vs. pure substrate ventricular tachycardia ablation: A case–control study

**DOI:** 10.3389/fcvm.2022.912335

**Published:** 2022-08-01

**Authors:** Sara Vázquez-Calvo, Paz Garre, Paula Sanchez-Somonte, Roger Borras, Levio Quinto, Gala Caixal, Margarida Pujol-Lopez, Till Althoff, Eduard Guasch, Elena Arbelo, José Maria Tolosana, Josep Brugada, Lluís Mont, Ivo Roca-Luque

**Affiliations:** ^1^Department of Cardiology, Cardiovascular Clinical Institute, Arrythmia Section, Hospital Clínic, Universitat de Barcelona, Barcelona, Spain; ^2^Institut d’Investigacions Biomèdiques August Pi i Sunyer (IDIBAPS), Barcelona, Spain; ^3^Centro de Investigación Biomédica en Red de Enfermedades Cardiovasculares (CIBERCV), Madrid, Spain

**Keywords:** ventricular tachycardia ablation, high-density mapping catheters, activation mapping, cardiac magnetic resonance, arrhythmic burden

## Abstract

**Background:**

Substrate-based ablation has become a successful technique for ventricular tachycardia (VT) ablation. High-density (HD) mapping catheters provide high-resolution electroanatomical maps and better discrimination of local abnormal electrograms. The HD Grid Mapping Catheter is an HD catheter with the ability to map orthogonal signals on top of conventional bipolar signals, which could provide better discrimination of the arrhythmic substrate. On the other hand, conventional mapping techniques, such as activation mapping, when possible, help to identify the isthmus of the tachycardia.

**Aim:**

The purpose of this study was to compare clinical outcomes after using two different VT ablation strategies: one based on extensive mapping with the HD Grid Mapping Catheter, including VT isthmus analysis, and the other based on pure substrate ablation.

**Methods:**

Forty consecutive patients undergoing VT ablation with extensive HD mapping method in the hospital clinic (November 2018–November 2019) were included. Clinical outcomes were compared with a historical cohort of 26 consecutive patients who underwent ablation using a scar dechanneling technique before 2018.

**Results:**

The density of mapping points was higher in the extensive mapping group (2370.24 ± 920.78 vs. 576.45 ± 294.46; *p* < 0.001). After 1 year of follow-up, VT recurred in 18.4% of patients in the extensive mapping group vs. 34.6% of patients in the historical control group (*p* = 0.14), with a significantly greater reduction of VT burden: VT episodes (81.7 ± 7.79 vs. 43.4 ± 19.9%, *p* < 0.05), antitachycardia pacing (99.45 ± 2.29 vs. 33.9 ± 102.5%, *p* < 0.001), and implantable cardioverter defibrillator (ICD) shocks (99 ± 4.5 vs. 64.7 ± 59.9%, *p* = 0.02).

**Conclusion:**

The use of a method based on extensive mapping with the HD Grid Mapping Catheter and VT isthmus analysis allows better discrimination of the arrhythmic substrate and could be associated with a greater decrease in VT burden.

## Introduction

Catheter ablation has become a standard treatment for ventricular tachycardia (VT) in patients with structural heart disease (1). Activation and entrainment mapping can be performed in only 30–40% of cases due to hemodynamically untolerated VTs. Substrate-based ablation based on the identification of the arrhythmic substrate through mapping during sinus or paced rhythm has been developed in recent years, initially to treat poorly tolerated infarct-related VT (2, 3). Nowadays, it has become established as the cornerstone for VT ablation in patients with structural heart disease. The main mechanism behind scar-related VT is the re-entrant circuit. This circuit is caused by the presence of a channel of border zone tissue with slow conduction surrounded by core scar tissue that connects to healthy tissue, leading to re-entry. These regions are also called conducting channels (CCs). These CCs can be accurately identified with the aid of electroanatomical maps (EAMs) obtained during ablation (2, 3). In these areas, reduced conduction velocity produces areas of delayed, fractionated electrical activity that can persist even after the inscription of the QRS complex (3, 4). Several studies have demonstrated the relation of these electrograms, so-called late potentials (LPs) or local abnormal ventricular activity (LAVA), with VT (5). Indeed, many authors have suggested that complete elimination of the VT substrate results not only in tachycardia non-inducibility, but also results in fewer VT recurrences on long-term follow-up (1, 6, 7).

This substrate approach strategy is based on the correct identification and posterior elimination of the entire arrhythmic substrate. The bipolar signal, especially in cases of fractioned and/or low-amplitude electrograms (EGMs) as LPs or LAVAs, depends on the electrode size, interelectrode spacing, and angle of the incoming wavefront to the mapping catheter (8, 9). Multielectrode high-density (HD) catheters have been developed to increase mapping resolution, allowing detailed anatomical characterization of the endocardial substrate (10). To overcome the dependence of the EGM amplitude on the wavefront orientation, a new high-density catheter with a grip shape was designed. This catheter (the Advisor™ HD Grid Mapping Catheter, Abbott Medical, United States) receives bipolar signals between each of its 16 electrodes, forming an extensive network of orthogonal signals. Through a dedicated algorithm (HD Wave Solution algorithm, Abbott Medical, United States), the highest peak-to-peak voltage among the two orthogonal signals of every dipole is selected. This grid catheter has been shown to facilitate the discrimination of LAVAs/LPs, providing a more detailed characterization of the arrhythmic substrate (11, 12), which is even more important in low-voltage areas.

This study aimed to compare the clinical outcomes of VT ablation guided by two different strategies: one based on high-density mapping with the HD Grid Mapping Catheter and induction and the other based on a scar dechanneling technique using the conventional bipolar HD Grid Mapping Catheter.

## Materials and methods

### Study population

This was a single-center, observational, prospective study that included all the consecutive patients who underwent VT catheter ablation with an extensive mapping method with the HD Grid Mapping Catheter in our center from October 2018 to December 2019 (group 1). This cohort was compared with a historical cohort of 26 consecutive patients who underwent ablation for VT from January 2013 to November 2018 using a scar dechanneling technique with an HD catheter without the ability to detect orthogonal signals (PENTARAY, Biosense Webster, Diamond Bar, California, United States) (group 2). The inclusion criteria were the presence of structural heart disease and episodes of sustained monomorphic VT. This study was carried out according to the guidelines of the Declaration of Helsinki and the deontological code of our institution. This study protocol was approved by the ethics committee of the hospital.

### Preprocedural evaluation

The number of events before the procedure was collected, as well as other clinical aspects, including the type of heart disease, antiarrhythmic treatment, and the New York Heart Association (NYHA) functional class. A cardiac ultrasound was performed on all the patients. Cardiac MR (CMR) with late gadolinium enhancement (LGE) was performed, if there were no contraindications [3 T scan in patients without an implantable cardioverter defibrillator (ICD) or 1.5 T with a wideband sequence in patients with ICD]. Manual segmentation of the left ventricle and right ventricle was performed. Automatic quantitative analysis of the substrate was executed using ADAS 3D software (ADAS 3D, ADAS 3D Medical SL) following a previously described protocol (13). In brief, the analysis was performed in 9 layers from the endocardium to the epicardium. A three-dimensional map was obtained for each layer. The LGE pixel signal intensity maps obtained from the CMR were projected onto each layer according to a trilinear interpolation algorithm and were color-coded. To identify the areas of scarring, an algorithm based on the intensity of the pixel signal was applied to characterize the areas of hyperenhancement, such as the core of the scar or the border zone, using 40 ± 5% and 60 ± 5% of the maximum intensity as a threshold, respectively.

### Ablation procedure

Procedures were performed under general anesthesia. Access to the left ventricle was achieved with a transseptal and/or retrograde aortic approach. Epicardial mapping was performed in cases when an epicardial origin of VT was suspected and in cases of failed endocardial ablation.

A substrate voltage map of the left ventricle was obtained during right ventricular (RV) pacing for better stability of the cardiac cycle using the HD Grid Mapping Catheter and the EnSite Precision Cardiac Mapping System (Abbott Medical, United States) in group 1 and the PENTARAY Catheter and the CARTO^®^ 3 system (Biosense Webster, Diamond Bar, California, United States) in group 2. Peak-to-peak amplitudes of 0.5–1.5 and < 0.5 mV were used to define the low-voltage zone and the dense scar zone, respectively. Abnormal electrogram features, such as “LAVA” (defined as local EGMs with split, fractionated, or high-frequency components) and LPs, were manually tagged. In both the groups, areas of scarring were confirmed with a contact-sensor ablation catheter (TactiCath SE, Abbott Medical in group 1 and SmartTouch, Biosense Webster in group 2) using 4 gr of contact as the cutoff point to avoid the overdetection of low-voltage areas (14). In both the groups, LGE-CMR postprocessed images (when available) were used to aid the ablation. In group 1, persistent scatterer interferometry (PSI) maps were visualized by the navigation system side by side. In group 2, PSI maps were merged with the substrate map as previously described (2).

#### Group 1

After voltage mapping and tagging of LAVAs and LPs, analysis of deceleration zones was performed by activation mapping during RV pacing. Automated annotation was performed at the offset of the latest local EGM component (last deflection algorithm, Abbott Medical, United States). After the delineation of slow conduction areas, the HD Grid Mapping Catheter was positioned in a potential area of the VT isthmus (slow conduction area and/or channel isthmus according to LGE-CMR images). VT was induced by programmed electrical stimulation (drive cycles of 600, 500, and 430 ms, up to triple extrastimuli to refractoriness or 200 ms). When VT was hemodynamically tolerated, activation mapping for diastolic and presystolic activities was performed. Entrainment mapping was performed at sites that demonstrated diastolic activity. In cases in which VT was not hemodynamically tolerated, the VT isthmus was defined as the area with a fast transition from good pace mapping (suggesting VT exit site) to poor pace mapping (suggesting VT entrance site), as described previously (15). Radiofrequency (RF) was delivered using an externally irrigated 3.5-mm tip ablation catheter with 45°C temperature control (irrigation rate of 26–30 ml/min), and a power limit of 40–50 W (60 W in septal areas and/or in cases of a deep substrate according to LGE-CMR PSI maps). The primary target for ablation was the central isthmus of VT according to activation mapping and/or pacemap mapping. After the VT isthmuses were targeted, substrate ablation was performed. The main targets were the deceleration zones and the entrances and exits of the defined conducting channels. Remapping with the HD Grid Mapping Catheter was performed to detect residual substrate. Additional RF lesions were delivered in areas with persistent LAVA and/or isolated LPs. The procedural endpoint was the abolition of LAVA and LPs, as well as the lack of VT inducibility at the end of the procedure.

#### Group 2

Substrate ablation was performed following a 4-step scar dechanneling technique, as previously described (2). The workflow involved the identification of CCs by EAM (and/or by LGE-CMR postprocessing model reconstruction) during sinus rhythm or RV stimulation. Isolated late potentials (ILPs) were manually tagged during mapping to define CCs inside the scar. Radiofrequency was delivered using an irrigated 3.5-mm tip ablation catheter with 45^°^C temperature control, power limit of 40–50 W, and irrigation rate of 26–30 ml/min (40 W and 17 ml/min at epicardium) at the CC entrance during sinus rhythm. Remapping was used to confirm the elimination of all the CCs and to check for residual ILPs. Residual ILPs identified by remapping were completely eliminated when possible. Programmed electrical stimulation (drive cycles of 600 and 400 ms, up to triple extrastimuli to refractoriness or 200 ms) was performed after substrate ablation, and any residual-induced VT was targeted. The procedural endpoint comprised the entrance of CC abolition, late potential abolition, and no VT inducibility at the end of the procedure.

### Ventricular tachycardia burden

The preprocedural burden of VT was defined as the number of VT episodes and ICD therapies within the last year until the day of the ablation procedure. The postprocedural burden of VT was established as the number of VT events during the 12 months after ablation.

A VT episode was defined as continuous VT for 30 s and/or a syncopal event or as VT that required an appropriate intervention for termination.

Implantable cardioverter defibrillator therapies were qualified and quantified by remote monitoring and outpatient device monitoring.

### Follow-up

The primary endpoint was VT burden reduction. The arrhythmic load was evaluated by analyzing the number of VT events (previously defined) and ICD therapies. Death during follow-up was categorized as either cardiovascular or non-cardiovascular. Among cardiovascular deaths, sudden deaths attributed to ventricular arrhythmia were recorded.

### Statistical analysis

Continuous variables are presented as the mean ± *SD* or median with an interquartile range, as appropriate. A *t*-test was used to compare the means of two variables. Categorical variables are expressed as the total number or percentage and were compared between groups using the chi-squared test or Fisher’s exact test to compare the VT burden before and after the procedure; we used the non-parametric Wilcoxon test for paired data.

Recurrence-free survival over time was calculated by the Kaplan–Meier method, and comparisons between the groups were performed with the log-rank test. All the analyses were performed with SPSS version 18.0 (SPSS, United States) and R software version 3.6.1 (R project for Statistical Computing; Austria). All the statistical tests were two-sided, and *p* < 0.05 was considered statistically significant.

## Results

### Baseline characteristics

During this period, 40 patients undergoing VT ablation met the inclusion criteria. Two patients were excluded due to complications related to the epicardial approach in which no mapping was performed. Therefore, 38 patients were prospectively included (group 1). This cohort was compared with a historical cohort of 26 consecutive patients. Both the groups were similar with respect to age and sex distribution, baseline left ventricular ejection fraction (LVEF), and antiarrhythmic therapy before ablation. The baseline characteristics of the patients are shown in Table 1.

**TABLE 1:** Demographic and clinical baseline characteristics of the population.

	Group 1	Group 2	*P*-value
Age, years	64.42 ± 12.00	66.12 ± 12.19	0.583
Male	94.7%	88.5%	0.358
Hypertension	68.4%	80.8%	0.272
Diabetes mellitus	42.1%	34.6%	0.546
Dyslipidemia	64.9%	73.1%	0.491
COPD	12.1%	19.2%	0.451
CKD (< 60)	17.9%	34.6%	0.160
Permanent AF	13.2%	3.8%	0.209
LVEF (%)	34.38 ± 11.02	35.64 ± 12.00	0.681
LVEDD (mm)	61.35 ± 11.15	59.00 ± 8.60	0.457
LVESD (mm)	45.24 ± 16.65	42.86 ± 12.77	0.664
NYHA-I	27.3%	38.5%	0.218
NYHA-II	69.7%	50.0%	0.218
NYHA-III/IV	3.0%	11.5%	0.218
Ischemic cardiomyopathy	68.4%	88.5%	0.063
Beta blockers	72.2%	92.3%	0.048
ACE inhibitor	61.1%	53.8%	0.567
Sotalol	19.4%	11.5%	0.404
Amiodarone	72.2%	65.4%	0.564
CMR preintervention	76.3%	76.0%	0.977
Total Scar by CMR (g)	9.19 ± 5.93	10.17 ± 6.59	0.631
Total BZ by CMR (g)	22.16 ± 8.43	20.64 ± 11.17	0.646
Epicardial scar by CMR (cm^2^)	91.30 ± 61.28	89.34 ± 60.35	0.922
Arrhythmic storm	26.3%	26.9%	0.957
Incessant VT	16.2%	7.7%	0.317
Episodes 1 year preablation	4.97 ± 8.96	2.31 ± 2.02	0.142
ATP 1 year preablation	5.89 ± 9.77	4.54 ± 8.15	0.582
Shocks 1 year preablation	2.64 ± 4.84	2.54 ± 4.32	0.933

COPD, Chronic obstructive pulmonary disease; CKD, Chronic kidney disease; LVEF, Left ventricular ejection fraction; LVEDD, Left ventricular end-diastolic diameter; LVESD, Left ventricular end-systolic diameter; NYHA, New York Heart Association; ACE, Angiotensin-converting enzyme; ATP, Antitachycardia pacing.

### Mapping and ablation procedure

Ventricular tachycardia ablation was performed with an epicardial or endoepicardial approach in 8.1% of patients in group 1 and 32.0% of patients in group 2 (*p* = 0.021) and exclusively by an endocardial approach in the remaining patients. The average mapping points were higher in group 1 than in group 2 (endocardium: 2370.24 ± 920.78 vs. 576.45 ± 294.46, epicardium: 1509.00 vs. 846.29 ± 536.60, *p* < 0.001). The percentage of induced VTs in which the diastolic isthmus was confirmed (by activation mapping and/or pace mapping) was higher in group 1 (92.17 vs. 57.05%, *p* < 0.001). The duration of the procedure (255.06 ± 46.67 vs. 195.55 ± 67.07 min, *p* < 0.001), fluoroscopy exposure (37.591 ± 15.14 vs. 20.304 ± 8.48 min, *p* = 0.000), radiofrequency exposure (1791.48 ± 780.70 vs. 1073.27 ± 605.40 s, *p* < 0.001), and the number of radiofrequency applications (63.28 ± 25.10 vs. 31.59 ± 14.05, *p* = 0.000) were greater in group 1.

Regarding the acute procedural endpoint of ablation, there was no difference between the groups. Major complications occurred in 2 patients in group 1 (pericardial effusion and femoral pseudoaneurysm) vs. 3 patients in group 2 [femoral hematoma, complete atrioventricular (AV) block, and ischemic stroke]. The ablation procedure parameters are given in Table 2.

**TABLE 2:** Procedural characteristics.

	Group 1	Group 2	*P*-value
Transeptal puncture	78.4%	96.0%	0.053
Epicardial access	8.1%	32.0%	0.021
Retroaortic access	68.6%	28.0%	0.002
Endocardial mapping points	**2370.24 ± 920.78**	**576.45 ± 294.46**	**0.000**
Epicardial mapping points	**1509.00**	**846.29 ± 536.60**	**0.000**
Procedure duration, min	**255.06 ± 46.67**	**195.55 ± 67.07**	**0.000**
RF application time, sec	**1791.48 ± 780.70**	**1073.27 ± 605.40**	**0.001**
RF application, *n*	**63.28 ± 25.11**	**31.59 ± 14.05**	**0.000**
Fluoroscopy duration, min	**37.59 ± 15.1427**	**20.30 ± 8.4769**	**0.000**
Number of induced VT	**1.68 ± 1.165**	**0.79 ± 1.062**	**0.004**
Number of VT target	**1.63 ± 1.060**	**0.38 ± 0.647**	**0.000**
Number of VT ablated	**1.53 ± 1.082**	**0.25 ± 0.532**	**0.000**
Complete LAVA abolition	77.4%	84.0%	0.538
No VT inducibility postablation	74.7%	72.4%	0.226

RF, Radiofrequency.

### Follow-up

**FIGURE 1:**
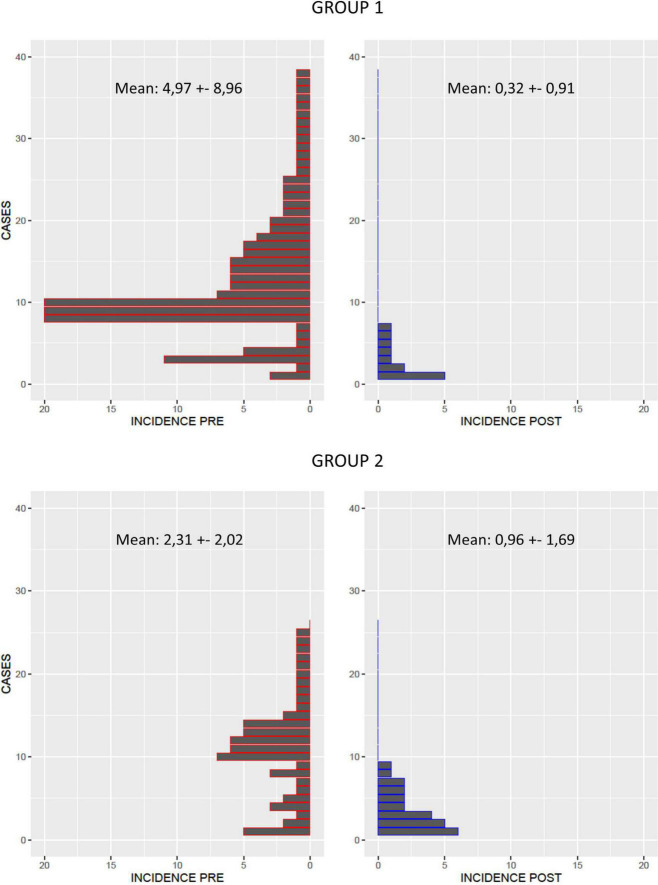
VT burden before (red) and after ablation (blue) in group 1 (extensive mapping) and group 2 (scar dechanneling). A reduction in VT was observed in both the groups, but the reduction was larger in the extensive HD mapping group.

**FIGURE 2:**
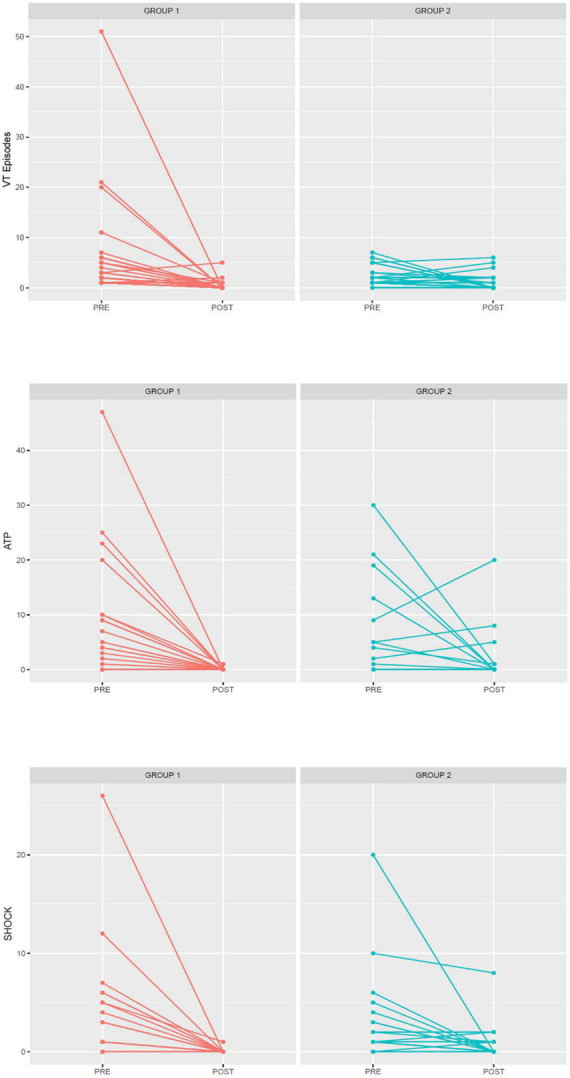
Differences in arrhythmic burden (VT episodes, ATP, and shocks) between pre- and postablation by group (group 1, extensive HD mapping, red; group 2, scar dechanneling, blue).

**FIGURE 3:**
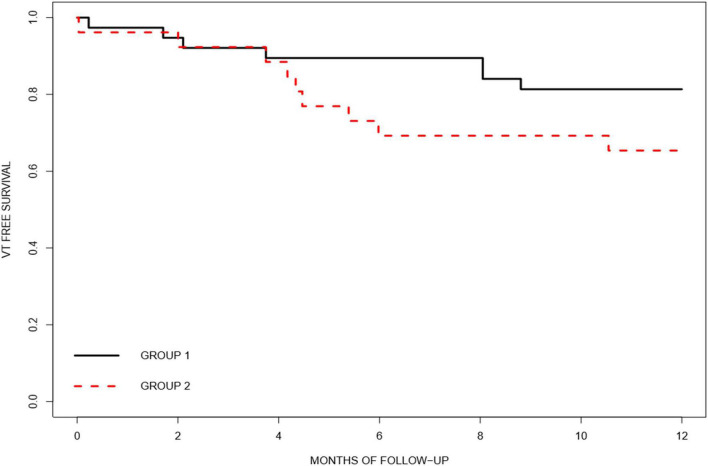
The Kaplan–Meier curve showing ventricular tachycardia-free survival after ablation by group (group 1, extensive HD mapping, black; group 2, scar dechanneling, red).

After 1 year of follow-up, a significantly higher reduction in the arrhythmic burden was observed in the extensive mapping group in terms of VT episode reduction (81.69 ± 7.79 vs. 43.46 ± 19.97%, *p* < 0.05), antitachycardia pacing (ATP) therapy reduction (99.47 ± 2.29 vs. 33.94 ± 102.46%, *p* < 0.001), and ICD shock reduction (99.00 ± 4.47 vs. 64.67 ± 59.87%, *p* = 0.02). Figures 1, 2 show the decrease in the VT burden in both the groups. In addition, a non-significant tendency toward a lower rate of VT recurrence (Figure 3) was also observed (18.4 vs. 34.6%, *p* = 0.142), with a hazard ratio of 2.032 (0.76–5.46) (*p* = 0.16). Only one patient from group 2 underwent a second VT ablation procedure. No patients from group 1 underwent a second VT ablation procedure. Death from any cause occurred in 3 patients in the HD mapping group and none in the historical cohort (*p* = 0.26). No deaths from a cardiac cause were observed.

## Discussion

We assessed the clinical efficacy of VT ablation with a method based on extensive HD mapping with catheters with the ability to analyze orthogonal signals (HD Grid Mapping Catheter) and induction vs. a scar dechanneling method with HD catheters that map only bipolar signals (PENTARAY). The major study findings were a higher substrate map density, a higher proportion of mapped VT, and a significantly larger reduction in VT burden (VT episodes and ICD therapies) in group 1.

Successful elimination of the arrhythmic substrate is based on the ability to create a detailed substrate map with identification of all the abnormal EGMs suggesting slow conduction. The resolution of electroanatomical mapping is determined by multiple parameters, including the mapping rhythm, vector of propagation, electrode size, interelectrode spacing, and filtering (8, 9). In this sense, several studies have shown that the use of multielectrode mapping catheters with smaller electrodes and interelectrode spacing can increase map resolution, enhancing the identification of surviving channels and macrore-entrant circuits (10, 16, 17). However, there is another key factor that determines the amplitude and shape of EGMs, i.e., the activation wavefront relative to the catheter orientation (18). Some studies have demonstrated, not only in animal models (19), but also in patients who underwent VT ablation (11), higher accuracy in scar detection with the use of catheters that are able to analyze orthogonal signals, with less beat-to-beat variation and better correspondence with histology (10).

In the present study, there was a clear difference in terms of HD mapping points in the HD mapping group in both the endocardium and epicardium, not only because of the different strategies. Higher-density mapping points have also been shown in other studies that compared the HD Grid Mapping Catheter with other HD mapping catheters (PENTARAY, Duo-Decapolar Catheter, etc.) (12). The HD Grid Mapping Catheter acquires simultaneous signals across orthogonal planes, achieving higher-density maps and providing a more thorough evaluation of the electrogram amplitude and direction. Therefore, by analyzing orthogonal signals at every single point, this catheter and its automatic algorithm enhance the identification of border zones and slow conduction areas (12, 16).

**FIGURE 4:**
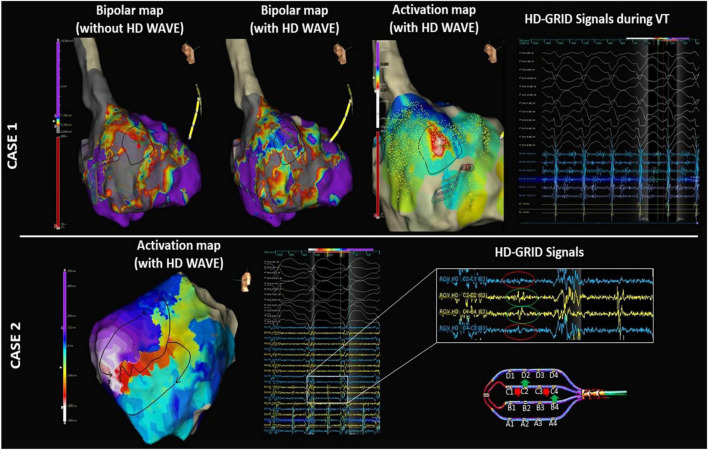
Electroanatomical maps (EAMs) of the left ventricle in 2 representative examples. Case 1: Left panel: high-density voltage map using only bipolar signals showing an extensive septal scar. Middle panel: the same map obtained using both the bipolar and orthogonal signals (HD Wave algorithm) shows “hidden” viable tissue in the upper part of this septal scar. Right panel: activation map obtained with the HD Grid Mapping Catheter and HD Wave algorithm shows mid-diastolic signals during VT. These mid-diastolic signals were located in the viable tissue only identified by the orthogonal signals during substrate mapping. Case 2: Activation map of VT obtained with the HD Grid Mapping Catheter and HD Wave algorithm. The importance of the orthogonal signals in relation to the activation wavefront is clearly illustrated: The conventional bipolar C1-C2 and C3-C4 (blue tracings) do not show any notable mid-diastolic potentials (red circles). In contrast, orthogonal signals in the same place (C2-D2 and B4-C4, yellow tracing) show clear mid-diastolic EGMs in the isthmus of VT (green circles). The VT activation map is shown in the left panel.

Similarly, more VTs were mapped in group 1. Detection of the diastolic isthmus in VT has recently been related to better long-term success (17). Currently, VT mapping is limited by poor tolerance of arrhythmia in many patients. However, the highly detailed substrate maps enabled the careful identification of potential slow conduction areas with the HD Grid Mapping Catheter before VT induction, which allowed us to perform limited VT mapping of the potential culprit areas in most of the patients. Because the map was limited to the region of interest, activation mapping could be performed in a short period of time, overcoming the problem of hemodynamic tolerance. In addition, the ability to detect orthogonal signals at every point increases the ability to detect diastolic low-voltage signals during tachycardia, as the wavefront direction is even more critical during tachycardia than during basal rhythm. A clear example of the relevance of orthogonal signals both in the substrate sinus map and VT activation map is shown in Figure 4.

Many studies have demonstrated that substrate ablation with the elimination of slow conduction areas (LAVAs, LPs, deceleration zones, etc.) is associated with better clinical outcomes (6, 12, 20, 21). As stated before, the properties of the HD Grid Mapping Catheter, including the capability of orthogonal signal detection, as well as the use of a specific algorithm (HD Wave Solution), reduce variability in electrogram characteristics associated with differential orientations relative to the propagating wavefront and allow selection of the highest-amplitude signal in each location. Concerning this matter, a small study (20) compared traditional point-by-point catheters vs. high-density catheters (PENTARAY) and found that the PENTARAY provided better discrimination of abnormal electrograms, without significant differences in VT inducibility after substrate ablation, but with a shorter radiofrequency time. Indeed, Proietti et al. (12) analyzed clinical outcomes after the use of different mapping catheters (HD Grid, PENTARAY, Duo-Decapolar, and point-by-point catheters) and found a reduction in ATP and/or significant appropriate shocks with HD catheters.

In our study, an ablation based on an extensive mapping with the HD Grid Mapping Catheter, compared to a scar dechanneling strategy with PENTARAY, resulted in a significant reduction in the overall VT burden at 1 year postprocedure, not only in ATP and appropriate ICD shocks but also in overall VT episodes. As mentioned above, due to the orthogonal signal mapping, more accurate substrate mapping and better discrimination of the VT diastolic isthmus can explain the better results obtained. Further larger and randomized controlled trials are needed to confirm these results.

## Limitations

This was an observational single-center study with the inherent limitations of such a study design. The retrospective cohort included a set of patients referred for ablation up to 5 years earlier than patients in the prospective cohort. During this time, some aspects, in addition to the use of a different mapping catheter, could have potentially contributed to the differences observed.

Another potential limitation is that although the main difference between the two groups was the use of different high-density mapping catheters, the ablation strategy was modified; in the PENTARAY group, a scar dechanneling technique was used in every patient, and in the HD Grid Mapping Catheter group, deceleration zone and VT isthmus analysis and ablation were included in the ablation strategy.

Epicardial access was more frequent in the historical group. In that period, any patient with some epicardial scar in the CMR, even patients with ischemic heart disease and also endocardial scar, underwent endoepicardial ablation. However, in the group with the extensive HD Grid Substrate Mapping Catheter, only epicardial access was performed in case of extensive epicardial scar or ECG suggestive of epicardial VT origin. To overcome some influence of larger epicardial scars in some of the groups with recurrence rate (as epicardial substrates are known to have higher recurrence rate), we have analyzed the amount of scar both in the endocardium and epicardium founding no differences between the groups; so, despite the lack of matching analysis, recurrence rate does not seem to be related with differences in scar amount.

Finally, the radiofrequency duration was also longer in patients treated with the HD Grid Mapping Catheter. However, the RF duration is similar to that in other published studies of the HD Grid Mapping Catheter (12) and could be related to the identification of slower conduction areas that ultimately need to be targeted.

## Conclusion

Ventricular tachycardia ablation strategy based on extensive mapping with the use of the HD Mapping Catheter with the ability to map orthogonal signals allows better definition of the arrhythmogenic substrate and better identification of the diastolic VT isthmus and may be associated with a lower arrhythmic burden on follow-up compared with conventional pure substrate-based strategy using the conventional bipolar HD Grid Mapping Catheter.

## Data availability statement

The raw data supporting the conclusions of this article will be made available by the authors, without undue reservation.

## Ethics statement

The studies involving human participants were reviewed and approved by the Ethical Committee of Hospital Clinic (CEIm). The patients/participants provided their written informed consent to participate in this study.
